# Lilium Tigrinum and Prolapsus Uteri

**Published:** 1889-06

**Authors:** Thomas G. Roberts


### LILIUM TIGRINUM AND PROLAPSUS UTERI.

THOMAS G. ROBERTS, M. D.

Some years ago Miss C., a brunette twenty-eight years of age, consulted me for relief from prolapsus uteri, that had severely troubled her for several years. She had been under regular treatment for a long time, and had been treated with pessaries, injections, tonics, etc., but, thus far, without securing the much-desired relief. She was much discouraged, and often felt like ceasing all efforts to recover her health; but, as she had never tried Homœopathy, she thought she would see if it could produce any better results than had been exhibited by the dominant school. She was so low-spirited that she could hardly keep from crying, and I have rarely seen one who looked so melancholic and forlorn. She was annoyed with a constant hurried feeling, as if she must immediately attend to important duties, and she manifested, in a marked degree, opposite and contradictory mental states.

Her greatest suffering was a dragging or bearing-down sensation, that extended from the chest and shoulders all the way down to the vulva; and this feeling was so intense that it seemed to her that all the pelvic viscera were being gradually forced through the vagina. There was a feeling that the abdominal and uterine regions needed support, and, to relieve the bearing-down sensation, she sometimes pressed with both hands against the vulva. In addition to the symptoms already given, she had, in the left ovary, a pain, running down. As every symptom was characteristic of Lilium tigrinum, that remedy was prescribed in the 30th potency, and the patient was requested to take a dose morning and evening for one week, and then report to me. At the end of that time a marked improvement was manifest; and, giving a placebo for ten days, the same remedy, in the 200th, was given at increasing intervals for about three months, when not a vestige of her trouble remained. Not long after she married, and is now the happy mother of three children; and, as I have been her physician ever since, I know that she never had a return of her ailment. No change was made in the dietetic or other habits of the patient, and no local measures of any kind were used. This was one of the first cases of chronic disease that I cured with the single remedy, and it made a great impression on my mind. Surely, nothing else is so curative as the simillimum; and cases like this ought to stimulate every disciple of Hahnemann to use the utmost care in the selection of the remedy, for, when the simillimum is found and rightly administered, the results that follow seem almost miraculous.

